# Complications of hypothermia: infections

**DOI:** 10.1186/cc11277

**Published:** 2012-06-07

**Authors:** Gregor Broessner, Marlene Fischer, Peter Lackner, Bettina Pfausler, Erich Schmutzhard

**Affiliations:** 1 Neurologic Intensive Care Unit, Medical University Innsbruck, Austria

## Background

Therapeutic hypothermia (TH) is a very elegant way of inducing short-term and long-term neuroprotection in various disease entities. It has become the standard of care after cardiac resuscitation with an impressive outcome improvement in prospective randomised trials. However, by a broader use of this sophisticated measure the critical care community has become aware of potential side effects limiting its effect on patient outcome. Among others, an increased rate of infections is observed under therapeutic hypothermia and controlled normothermia. The pathophysiological considerations by which TH increases infectious complications comprise reduced inflammatory response and suppression of leukocyte migration and phagocytosis. All together, these observations justify a high vigilance towards infectious manifestations if temperature modulation measures, namely therapeutic hypothermia and controlled normothermia, are used in critical care patients.

## Pathophysiological considerations about hypothermia and infections

Intriguing data derived from animal models showing a potent neuroprotective effect in various disease entities induced by hypothermia gave way to a broader use of this method in humans. Thus, therapeutic hypothermia has become the standard of care after cardiac resuscitation as studies demonstrated its strong neuroprotective effect and neurologic outcome improvement [1,2]. It is now recommended by the European Resuscitation Council and the International Liaison Committee on Resuscitation in cases of comatose adults with spontaneous circulation after out-of-hospital cardiac arrest (OHCA) [3]. However, in indications other than resuscitation, such promising results could not be achieved in prospective trials shifting the scientific focus on possible side effects of TH [4-6]. Rewarming injury, shivering, electrolyte dysbalance, pharmacological and pharmacodynamic alterations, cardiovascular effects including arrhythmia, insulin resistance and infections have recently been attributed as limitations occurring in a dose-dependent fashion under TH [7]. Taken together, maximal reduction of these side effects should be a treatment goal if dealing with temperature control measures irrespective of the target temperature. Today, infectious complications are thought to be one of the major contributors limiting the effects of hypothermia [7-12]. Thus advancing diagnostic approach, prevention and treatment of these infectious complications is a great concern of the scientific critical care community and need to be addressed in future prospective trials. In various studies enrolling patient populations suffering from such different diseases as traumatic brain injury, ischaemic stroke or resuscitation post cardiac arrest, an increased rate of infections under TH was observed [4,6,11]. Whether this negative effect has to be attributed to a specific cooling measure remains under debate; however, this hypothesis is unlikely as increased infections are found under both endovascular and surface cooling measures.

The biological interpretation of the pathophysiological backgrounds is challenging as temperature modulation inhibits various inflammatory responses on different levels that are only partly understood today [7,8]. Hypothermia impairs the secretion of proinflammatory cytokines and suppresses leukocyte migration and phagocytosis [7,8]. Recently it has been speculated that hypothermia may induce insulin resistance leading to hyperglycaemia possibly promoting infection onset [8,13].

However, increased rate of infections has also been observed not only under TH but also under endovascularly controlled prophylactic normothermia in patients with severe cerebrovascular disease [10,14]. A significant increase of infectious complications was observed in the endovascular treatment group although TH was strictly avoided. Importantly in this study from our group, analysis of the inflammatory parameters revealed a significant increase of C-reactive protein (CRP) in the prophylactic normothermia group whereas procalcitonin (PCT) and white blood cell count were not elevated [10]. This is a crucial point as it might indicate that temperature modulation may influence the prognostic value of inflammatory parameters [10].

In a retrospective review by Mongardon and coworkers including 421 patients being treated after cardiac arrest, in 281 patients (67%) an infectious complication was diagnosed [11]. Pneumonia was the most frequent, followed by bloodstream infections and catheter-related infections [11]. Gram-negative bacteria were the most frequently isolated infectious germs, but the main pathogen detected was *Staphylococcus aureus *[11]. The high rate of reported pneumonia raises the question of whether intubation at an early stage should be considered in patients under TH to minimise the risk of aspiration.

## Is this surcharge too much and how can we minimise it in clinical routine?

Hospital-acquired infections lead to secondary injury in patients and are responsible for a considerable cost increase especially in critical care patients [15,16]. An increased rate of infections under controlled normothermia and therapeutic hypothermia has been described in patients suffering from ischaemic stroke, traumatic brain injury, spontaneous subarachnoid haemorrhage, post resuscitation and intracerebral haemorrhage [6,9,10]. If this observation is associated with significantly impaired outcome or even mortality is under debate [6,9-11]. However, there is general consensus that infections lead to prolonged ICU treatment, secondary injury and lastly to cost increase with significant global economic burden [15]. Therefore, the critical care community has to find effective strategies to minimise the risk of infection complications.

Temperature modulation by any means has to be combined with a standard operation procedure including: routine microbiological surveillance including blood, urine, respiratory specimen work-up; radiological pneumonia surveillance; daily monitoring of inflammatory parameters (CRP, PCT, leukocytes); routine check of catheter insertion sites and timely catheter replacement; avoidance of hyperglycaemia (under TH) and hypoglycaemia (while rewarming!); and monitor performance of cooling device as a high cooling power/rate might indicate fever even if the body core temperature is normal or even <36°C.

## Conclusion

These observations justify a high level of vigilance towards infectious manifestations if temperature modulation measures, namely therapeutic hypothermia and controlled normothermia, are used in critical care patients. Whether, at all, the early use of antibiotics in case of suspected infections under TH is justified has to be addressed in future prospective trials.
